# J.G.C. Lehmann's 'Botanical Observations' of 1818 on Coldenia, Colsmannia, Cynoglossum, and Omphalodes (commented translation)

**DOI:** 10.3897/BDJ.2.e1064

**Published:** 2014-04-22

**Authors:** Hartmut H. Hilger, Theodor C. H. Cole, Federico Selvi

**Affiliations:** † Freie Universität Berlin - Systematic Botany and Plant Geography, Berlin, Germany; ‡ Dept. Agrifood Production and Environmental Sciences (DISPAA), Sect. Soil and Plant Sciences, Botanical Laboratory, University of Florence, Firenze, Italy

**Keywords:** Boraginaceae, taxonomy, legacy literature republishing, translation, markups

## Abstract

An annotated English translation of a German early 19th century text including Latin diagnoses is presented with a high-quality scan of the original publication and direct links to the cited pages with taxon and literature citations (including TL-2 entries).

## Introduction

Lehmann JGC (1818) Botanische Beobachtungen. *Coldenia*, *Colsmannia*, *Cynoglossum*, *Omphalodes*. Der Gesellschaft Naturforschender Freunde zu Berlin Magazin für die neuesten Entdeckungen in der gesammten Naturkunde, Vol. 8 (1814–1818): 91–100, with 4 Figures (Plates IV–VII) [Apparently in the printed paper the plates referred to as IV to VII in the text were misnumbered I to IV, and the numbering corrected by hand at least in the attached copy and the online version from Göttingen [Lehmann 1818]. For a pdf of the original publication see Suppl. material 1.

Many contributions on Boraginaceae by German botanists up to the late 20th century were published in discontinued serial publications and have rarely if ever been cited in modern scholarly literature. Unawareness of such previous studies may lead to loss of valuable information and avoidable misunderstandings. A paper by Dandy and Stearn (1961), "What is *Cynoglossum lusitanicum* L.", is a particularly good example, as the topic had – with similar results as to the assignment of *Cynoglossum lusitanicum* to the genus *Omphalodes* – already been discussed by Lehmann in 1818, not cited in that article. When discussing this apparently language-related issue with other taxonomists, the suggestion was made to make these older papers available in English. We accordingly are here starting a series of republications of such valuable older literature. To improve readability, we translated the text, which is written in a 200-year-old German, not literally but freely whenever helpful for better clarity, using also Stearn 1992. In Lehmann's text we checked and completed his citations [in square brackets], linking them to online resources with the original literature, as available. If necessary, we applied current names based on recent phylogenetic investigations of the mentioned taxa. Plant names in footnotes are cited in conformity with international standards, adapted from IPNI. In the references we added links to the publication corresponding to each, with standard abbreviation, from the online version of Stafleu and Cowan 1979 (onward): "Taxonomic Literature II (TL-2): Taxonomic Literature: A selective guide to botanical publications and collections with dates, commentaries and types (Stafleu et al.)" at the  Smithsonian Institution Libraries.

## Translation [additions and comments by the authors in square brackets; portions in navy blue color are those that were translated from Latin]

### Coldenia

This genus belongs to the Pentandria Monogynia among the nutlet-bearing Asperifoliae^[1]^, its closest relative being the genus *Heliotropium*. Linné [Linnaeus 1753: 125] grouped it with his Tetrandria Tetragynia, and it has been retained in that position by most botanists, even by Willdenow^[2]^ and Persoon^[3]^. Tracing back to Linné's first editions of his Species Plantarum reveals though that he had not had the opportunity to adequately investigate this plant [*Coldenia procumbens*] as he specifically states that “Others may study the fruiting on live plants, I did not see it achieved”.

Gaertner [Gaertner 1788], in his work “De fructibus et seminibus plantarum Vol. 1. p. 329”, assigns to this genus four stamens and a style^[4]^ with two stigmas. Jussieu [Jussieu 1789] (Genera Plant. pag. 130) followed Linné’s generic characters but adds the question “does Coldenia rather have five stamens?”, especially because he had received a second species which according to Dombey’s affirmation displayed a calyx as well as a corolla with five incisions and thus needed to be classified with Pentandria Monogynia. The plant he refers to is identical to the one described by Dr. Persoon [Persoon 1805] (Synopsis plant. 1. pag. 157) as *Tiquilia dichotoma* – being *Lithospermum dichotomum* of the Flora Peruviana [Ruiz & Pavón] Vol. 2 pag. 5. tab. 111 [fig. c].

The habitus of the plants clearly indicates that the genus *Tiquilia* belongs to *Coldenia*^[5]^. The affiliation of *Coldenia* to the nutlet-bearing Asperifoliae is evident from a comparison with its next of kin *Heliotropium*, and I would consider *Coldenia procumbens* as only an a b n o r m a l species, if I had not personally seen pentamerous and pentandrous flowers on several plants collected in Guinea. Only the lowest flowers mostly lacked the fifth part in calyx and corolla as well as the fifth stamen, but all were monogynous. – In no case did I find 'bilocular nuts' which had also been reported for *Coldenia procumbens*, but rather in every case I identified four distinct, basally connate nutlets^[6]^, two of which were sometimes not fully developed.

[**1**] Boraginales (or Boraginaceae s. l. sensu APG III, Angiosperm Phylogeny Group 2009).

[**2**] No year indicated, but most probably Willdenow 1798: 712.

[**3**] No year indicated, but most probably Persoon 1805: 1.

[**4**] Lehmann, to designate the style, used the now obsolete term “Staubweg”, literally “dust way”, i.e., pollen thoroughfare or stylar transmission canal.

[**5**] Richardson (1976), Richardson (1977) again separated *Tiquilia* from *Coldenia*. Molecular phylogenetics prove *Coldenia* to be a member of Cordiaceae (Gottschling et al. 2005) and *Tiquilia* of Ehretiaceae (Weigend et al. 2013).

[**6**] Lehmann apparently used seed ("Saamen") for the dispersal unit and nut or nutlet ("Nuss", "Nüsse") for the structural/morphological unit.

### Colsmannia [Lehm.] (new genus)

*Natural character.*

CAL[YX]. Perianth monophyllous [synsepalous, fused], five-parted, campanulate, petaloid [showy], very large, persistent, with pentagonal base: lobes ovate-lanceolate, patent, acute at the apex, longer than corolla.

COR[OLLA]. With a single petal [sympetalous, fused], tubular-campanulate, shorter than calyx. Tube cylindrical. Limb tubular-ventricose, five-toothed. Throat bare, open.

STAM[ENS]. Filaments five, somewhat fleshy, inside corolla tube. Anthers subulate-sagittate, united in a pyramidal fascicle, free at base, erect, as long as filaments.

PIST[IL]. Ovules four. Style filiform, longer than corolla. Stigma obtuse.

PER[ICARP]^[7]^. Absent. Calyx swollen, enclosing seeds at the bottom.

SEM[EN – SEED]. Nutlets four, two opposite ovate, triangular, bone-like, shiny, smooth, fixed to the receptacle, perforate at the base, two often aborted.

 

*Essential character.*

Calyx five-parted, petaloid, very large, pentagonal at base. Corolla cylindrical-campanulate, shorter than calyx. Throat bare. Anthers hastate, free. Nutlets four, triangular, ovate shiny bone-like, perforate.

*Obs. 1)* Similar to Onosma especially as to corolla, but differing in: 1) calyx with pentagonal base, petaloid, longer than corolla, with lobes ovate-lanceolate, patent. 2) Anthers with a free base, in no way connected. 3) Nutlets with a perforate base.

*Obs. 2)* The species that shows these generic characters was collected in the Orient by Tournefort, if I’m not mistaken.

[**7**] Apparently Lehmann did not recognize that “nutlets” are parts of the fruit.

### *Colsmannia flava* [Lehm.]^[8]^ Plate IV. [Fig. 1]

Root brown, white inside, woody in the upper part, apparently perennial. Stems several, erect, simple, half-foot and more [>15 cm], like the whole plant very densely covered with yellow, appressed, soft hairs, thus the species name “flava”. Leaves perfectly entire, sericeous, obovate-lanceolate, somewhat obtuse, attenuate at base; the lower ones petiolate, the upper ones sessile, alternate. Inflorescence: terminal raceme. Flowers with pedicels, pendulous before anthesis, eventually erect; the lower ones with lanceolate bracts longer than pedicels. Dry calyx [v.s. = vidi siccum, i.e. = on herbarium material] colored, pale-yellow, sericeous, sometimes four-parted; fourth lobe two times larger than the remaining ones, with bifid apex. Corolla yellow, with subpubescent external face. Style purplish. Seeds are nutlets. Dry material seen.

Fig. 1

Fig. *a.* shows a dissected flower from inside.

Fig. *b.* two mature nutlets in natural size and position.

Fig. *c.* a single nutlet.

Fig. *d.* a cross section through a nutlet.

Fig. *e.* the lower part of (d) from below.

This genus is distinct in the first place from all other members of the family Boraginaceae or Asperifoliae by its petaloid calyx. As I have already mentioned, it most closely resembles the genus *Onosma* [Linnaeus 1762: 196], and within it, *Onosma sericeum*^[9]^. The genus *Triplaris*^[10]^, a tree in the Dioecia Decandria, has a rather similar calyx, except that, same as the corolla of that plant, it only has three parts.

I have named this genus after my revered friend, Professor Colsmann from Copenhagen; already well known to all botanists by his exemplary description of the plants belonging to *Gratiola* collected by Dr. König; and even more so by his vast knowledge of all domains of natural sciences and by the rare largesse by which he grants access to his excellent botanical, entomological, and mineralogical collections to anyone desiring to educate himself; a forthcoming that earned him wide-ranging reverence and affection.

[**8**] *Colsmannia* is now treated as synonym of *Onosma*. *Colsmannia flava* is actually *Onosma flava* (Lehm.) Vatke ex Boiss., Fl. Orient.: 186. 1875 (urn:lsid:ipni.org:names:119655-1), but apparently no DNA phylogeny is available for confirmation.

[**9**] *Onosma sericea* Willd., Sp. Pl. 1: 774. 1798 (urn:lsid:ipni.org:names:119807-1).

[**10**] *Triplaris* Loefl. (Polygonaceae).

### Cynoglossum - Omphalodes

These two genera markedly differ in habit and especially by the strikingly different aspects of their seed, which in the case of *Omphalodes* one would hardly call nutlets, were it not in order to include them among the nutlet-bearing Asperifoliae. Initially separated, they later were reunited by most botanists. At first, I would like to make some general remarks concerning various species of the genus *Cynoglossum*, to then present a monographic review of the *Omphalodes* species.

*Cynoglossum lateriflorum*^[11]^. Lamarck [Lamarck 1786] Dict. enc. Vol. 2 pag. 239. no. 10, and *Cynoglossum lineare*^[12]^ Ruiz. et Pavon. Fl. Per. Vol. 2. pag. 6 [Ruiz and Pavón 1799] are one and the same plant. The later name has therefore to be deleted from the system.

*Cynoglossum angustifolium*. Willd. [Willdenow 1798] Sp. plant. T. 1. P. 2 p. 763^[13]^, *Cynoglossum emarginatum*. Lamarck [Lamarck 1792] Illustr. Vol. 1. p. 400. no. 1799^[14]^ and *Cynoglossum racemosum* Schreb. [Schreber 1767] in Nov. Act. Nat. Curios. T. II. [in fact: III] [see also *Cynoglossum cristatum* Schreb.] Pag. 475^[15]^ also are one and the same plant. Schreber’s (e a r l i e r) description slightly differs only with respect to the nutlets from that of Willdenow. I know with certainty that Lamarck’s plant also belongs here because I saw original specimens during my stay in Paris; and all three are assigned the same synonym of Tournefort ["*Cynoglossum orientale minus, flore campanulato caeruleo*”, Tournefort 1703: 7]. The name given by Schreber seems to have been completely ignored by recent botanists [e.g., Lamarck 1786].

*Cynoglossum cristatum*. Schreber [Schreber 1767] in Nov. Act. Nat. Curios. Vol. III. pag. 476^[16]^ is the very plant that was (l a t e r) described by Lamarck [Lamarck 1792] 400, no. 1800, and others under the same name. Also in this case, it appears that Schreber's description has been overlooked.

*Cynoglossum echinatum*. Thunberg [Thunberg 1794] Prodr. Fl. Capens. Pag. 34^[17]^, and *Myosotis cynoglossoides* Lamarck [Lamarck 1792] Illust. Vol. 1. pag. 396 no. 1778 again are one and the same plant. Thunberg [Thunberg 1806] later described this species in detail in the 5^th^ [in fact: 3^rd^!] part, pag. 43. [in fact: 48!] of the first volume of Schrader’s neuem Journal für die Botanik 1806. Lamarck’s description of the seeds is very accurate; I agree that this plant should be placed in *Myosotis*^[18]^.

*Cynoglossum hirsutum*. Thunb. [Thunberg 1794: 34] l.c. and *Cynoglossum lanceolatum* Forsk. [Forsskål 1775]^[19]^ Descript. p. 41 also do not differ from each other.

*Cynoglossum cheirifolium*. Linn. [Linnaeus 1753: 134]^[20]^ and *Anchusa lanata*. Linn. [Linnaeus 1759: 914]^[21]^ are also one and the same plant. Vahl had noted this when studying Linnaeus's herbarium, and Hornemann [Hornemann 1813] included that note in the Enumerat. Plant. Hort. Bot. Hafn. [in fact: Hortus regius botanicus hafniensis Vol. 1 (1813)] Vol. 1. pag. 177. The plant that Willdenow [Willdenow 1809] described as *Cynoglossum cheirifolium* in his Enumeratio pl. hort. Bot. Berol. Vol. 1 pag. 180 is, according to Hornemann (l. c. [a.a.O. = am angegebenen Ort]), a different species, and the described characteristics seem to support that view. However, the study of specimens from the local [Berlin] botanical garden convinced me that this is not true and I suspect that, only due to a slip of the pen of Willdenow was the calyx said to be larger (or better, longer) than the corolla.

*Cynoglossum fulvum*. Rudolphi [Rudolphi 1800] in Schrader’s Journal für die Botanik 2^nd^ vol. 1799^[22]^ 4^th^ Stück [part] pag. 279, *Cynoglossum clandestinum*. Desfont. [Desfontaines 1789] Fl. Atlant. T. 1 pag. 159. tab 42, and *Cynoglossum officinale* Brot. [Brotero 1804] Fl. Lusit. I. pag. 295 are all identical (compare Hoffm. et Link. [Hoffmannsegg and Link 1811] Flore portugaise I. pag. 190). Dr. Persoon [Persoon 1805] falsely believes (Synops. plant. I. 159) that Rudolphi’s plant belongs to the *Buglossa*^[23]^ and is closely related to *Anchusa italica*. I would like to parenthetically add that *Anchusa italica* Retz. [Retzius 1779] Observ. bot. Fasc. I. pag. 12 is not different from *Anchusa paniculata*. Ait. [Aiton 1789] Hort. Kew. ed. I. [vol. 1] pag. 177 and that *Anchusa officinalis* of [Desfontaines 1789]^[24]^ Fl. Atlant. I. pag. 157 also belongs to this group.

*Cynoglossum Dioscorides*. Villars [Villars 1787]. Fl. Delph. [Dauphiné] Vol. 2 pag. 457. is neither a variant of *Cynoglossum officinale* as Willdenow [Willdenow 1798] sp. plant. T. I. P. 2. pag. 760 [Spec. Pl. ed. 4 2(1)] claims nor is it identical with *Cynoglossum montanum*^[25]^ or *Cynoglossumsylvaticum* [Haenke 1789: 77] as is commonly believed. I own a specimen that Villars himself communicated to me, which totally differs from *Cynoglossum officinale* as well as from *Cynoglossum sylvaticum*.

*Cynoglossum laevigatum*. Linné [Linnaeus 1774]^[26]^.

The famous Pallas [Pallas 1771: 486] described this plant under the name *Rindera tetraspis* in the first volume of his “Reise” (append. No. 100 [p. 486] tab. 100 [in fact: 101]) but later on, in his Flora Rossica [Pallas 1789: 96] listed it as *Cynoglossum Rindera*, which the younger Linné [Linnaeus fil. 1782] (Supplement pag. 130)^[27]^ had originally proposed. The latter author must have considered it to be different from the *Cynoglossum laevigatum* of his father, because he chose to describe it under a d i f f e r e n t name, in contrast to Willdenow's [Willdenow 1798] (Spec. plant. T. I, P. 2. p. 763) e r r o n e o u s quote. Now this *Cynoglossum laevigatum* has been assigned by Schultes [Schultes 1809: 30], along with some other species [of the genus *Cynoglossum*] to a separate genus newly named *Mattia*^[28]^. At a first glance, *Cynoglossum laevigatum* along with *Cynoglossumlanatum* and *Cynoglossumumbellatum* seem indeed to make up an own genus, considering the differences in flowers and seeds. But if one compares the flowers and seeds of *Cynoglossum glastifolium*, *Cynoglossumangustifolium*, *Cynoglossumstamineum*, *Cynoglossumcristatum*, *Cynoglossumlateriflorum* and some other less known species, which Mr. Schultes [Schultes 1809] perhaps did not have the opportunity to compare, the transition to the remaining *Cynoglossa* is very striking, and I thus believe that these species may not be considered to constitute a genus of their own. If one persists in separating them, they should at least be left under the earlier and more commonly known generic name [*Rindera*].

*Cynoglossum lusitanicum*. Linn. [Linnaeus 1762] Sp. plant. ed. I. p. 293 [in fact: 193].

This name has been applied to no less than five, perhaps even more completely different plants. Linnaeus's *Cynoglossum lusitanicum*, as we learn through Link [Link 1806], according to Smith (the owner of Linnaeus's herbarium) is a Siberian plant (compare Schrader’s Neues Journal für die Botanik 1. Band 1806. 3rd Stück [piece] pag. 183 [in fact: 139]^[29]^) which, as we also know from Link, does not occur in Portugal^[30]^.

*Cynoglossum lusitanicum*. Vahl [Vahl 1791] Symb. bot. 2. pag. 34.

This plant, which I will describe further down as *Omphalodes amplexicaulis* does not grow wild in Portugal either (compare Brot. [Brotero 1804] Fl. lusit. Vol. I. pag. 296) and is also apparently extinct in the gardens. The specimen stored in Vahl’s collection is from the Botanical Garden of Madrid, edited by Dr. Bernades in July 1760 and designated with the Tournefortian [Tournefort 1700b: 140] name *Omphalodes lusitanica elatior cynoglossi folio*. I do not understand how plants described as

Cynoglossum with leaves clasping the stem, cordate and smooth at the margins. Vahl l. c. [Vahl 1791: 34]

could have been put together with the Linnaean [Linnaeus 1762: 193]

Cynoglossum with linear-lanceolate, rough leaves.

*Cynoglossum lusitanicum*. Brot. [Brotero 1804] Fl. lusit, I. pag. 296 is entirely different from the above-mentioned plants, and has been described by Professor Link [in Hoffmannsegg and Link 1811] as *Omphalodes nitida* Flore portugaise I. pag. 192–94, which is gorgeously depicted in tab. 25. Willdenow [Willdenow 1809] listed this species in his Enumeratio plant. Hort. Berol. Vol. I. pag. 181 under the name *Cynoglossum nitidum*.

*Cynoglossum lusitanicum*. Lamarck [Lamarck 1786] Dict. enc. Vol. 2. pag. 239 I do not consider different from *Cynoglossum lusitanicum* Brot., or from *Omphalodes nitida* Link. Lamarck’s description, more than his diagnosis, is generally appropriate, while not totally correct. What I have seen in the Paris collections as *Cynoglossum lusitanicum* was identical with *Omphalodes nitida*. Prof. Link considers Lamarck’s plant as a different species (comp. [Hoffmannsegg and Link 1811] Flore portugaise I. pg. 195).

*Cynoglossum lusitanicum*. Miller [Miller 1768] Dict. no. 6 is a mere variety of *Omphalodes linifolia*.

Finally, the Abbée [sic] Fortis [Fortis 1771] in his Osservazioni sopra Cherso ed Osero pag. 68. describes a *Cynoglossum lusitanicum*, which is certainly different from all other plants bearing that name. Because his description is short and little known, it may be appropriate to repeat it here: “Plant one foot [30 cm] tall. Root biennial, woody. Leaves ovate-lanceolate, absolutely entire, villous, ciliate towards base. Stems slightly striate, rough. Flowers small, opposite to leaves, pale blue. Seeds small, muricate. Fortis l.c. [locus citatus = place cited before]”.

 

Omphalodes Tournefort. [Tournefort 1700a] tab. 58. Gärtn. [Gaertner 1788] tab. 67. f. 3 C

*Essential character.*

Calyx deeply divided in five parts. Corolla rotate, closed at throat by arch-like scales. Nutlets four, depressed (concave), obliquely overtopping style, with membranous margin, calathiform [cup-shaped].

1) *Omphalodes nitida. Hoffm. et Link *[Hoffmannsegg and Link 1811].

Omphalodes with oblong-lanceolate leaves, veined, glabrous and shiny above, pubescent below, the lower ones long-petiolate, the upper ones sessile.

Omphalodes nitida. Hoffm. et Link [Hoffmannsegg and Link 1811]. Fl. Portugaise I. p. 194

*Cynoglossum nitidum*. Willd. [Willdenow 1809]. Enumerat. I. p. 181

*Cynoglossum lusitanicum*. Broter. [Brotero 1804]. Fl. Lusit. I. p. 296

*Cynoglossum lusitanicum*. Lamarck [Lamarck 1786]. Enc. Bot. Vol. 2. p. 239

*Omphalodes lusitanica cynoglossi folio*. Tournef. [Tournefort 1700b] Inst. rei herb. p. 140.

*Descript*[*ion*]. Hoffm. et Link [Hoffmannsegg and Link 1811]. l. c. p. 192–195.

Lamarck [Lamarck 1786] l. c. [239]

*Icon.* Hoffm. et Link l. c. [Hoffmannsegg and Link 1811]. tab. 25.

Grows in Portugal in moist, shady, naturally forested areas. [symbol:] perennial (seen alive) [*v. v.* = vidi viva].

In our greenhouses this nice plant flowers at the beginning of May.

 

2) *Omphalodes cornifolia*. (to me)

Omphalodes with long-petiolate radical, ovate-cordate, acuminate, veined leaves, the cauline ones subsessile, lowest ones lanceolate, the highest ones ovate, racemes solitary with many flowers.

*Cynoglossum cappadocicum* Willd. [Willdenow 1798] Sp. plant. T. I P. 2 pag. 767.

*Cynoglossum omphalodes*. β Lamarck [Lamarck 1786] Enc. Bot. V. 2. p. 239

*Omphalodes orientalis corni folio*. Tournefort [Tournefort 1703] Cor. p. 7.

*Descript*[*ion*].

Plant of about seven and a half inches [19 cm]. Stems erect, filiform, pubescent-hairy. Leaves with entire margins, subglabrous above, hispidulous under the lens [when magnified], glabrous below, sparsely pilose, with alternate, prominent veins, farinaceous; the radical ones very long-petiolate, ovate-cordate, acuminate, 2–4 inches [5–10 cm] long, 1–2 inches [2.5–5 cm] wide; lower cauline ones sessile, oblong-lanceolate, acute on both sides, short-petiolate, less than one inch [< 2.5 cm]; upper or floral ones of 1 inch [2.5 cm], sessile, ovate, acute. Flowers alternate set apart, pendulous before anthesis, erect after flowering, in terminal, solitary, elongated, loose racemes. Pedicels capillary [very thin], one inch and more [>2.5 cm], sparsely hairy. Calyces hairy especially towards base, five-parted; lobes ovate, acuminate.

**Figure 2.**
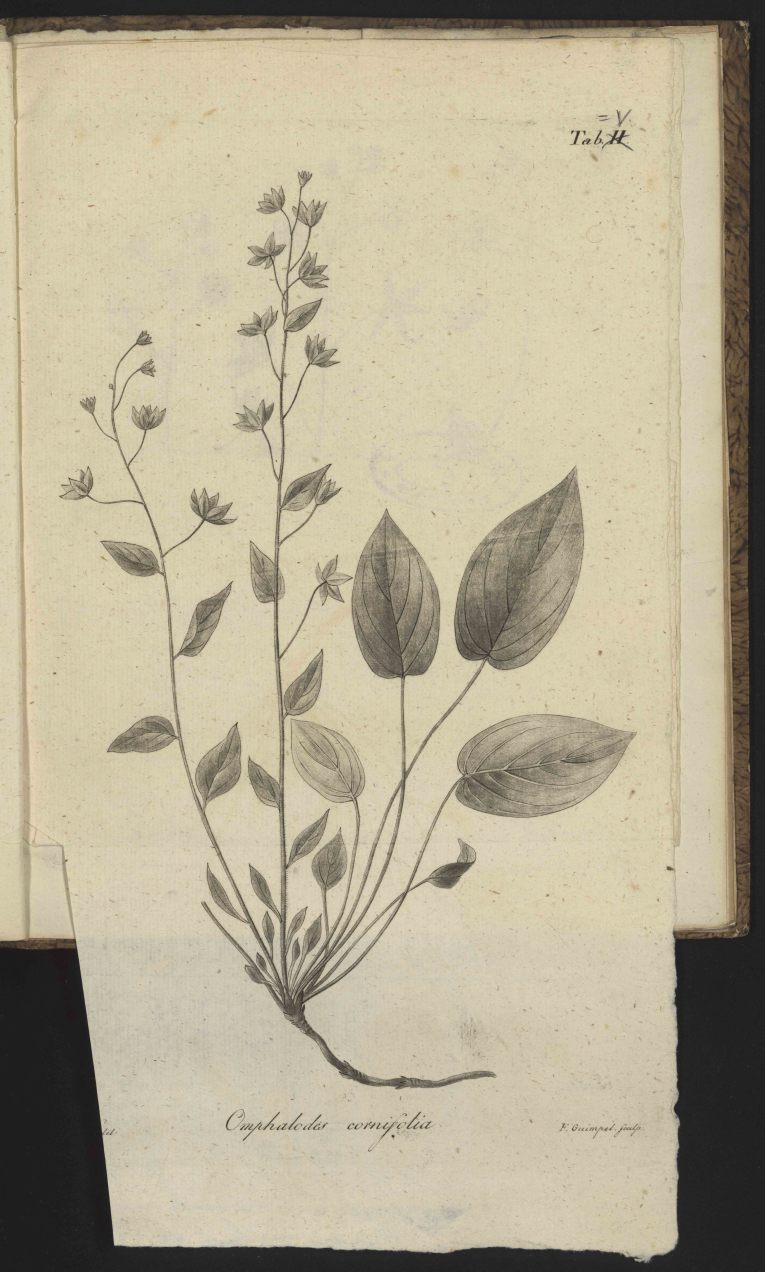
Facsimile of Lehmann 1818 pl. 5.

*Icon.* Plate V. [Fig. 2]

Growing in Cappadocia [Turkey]. [symbol:] perennial herb (dry material seen)

Fig. 2

 

3) *Omphalodes verna*. *Moench*.

Omphalodes with cordate, stem-clasping leaves, the lower ones strongly obtuse, the upper ones slightly acute, glabrous, smooth at the margins, racemes erect with several flowers.

Omphalodes verna Moench [Moench 1794]. Methodus. pag. 420.

*CynoglossumOmphalodes*. Linn. [Linnaeus 1762] Sp. Plant. 1. p. 193.

*Descript*[*ion*]. Scopol. [Scopoli 1771] Fl. Carn. ed. 2. 1 pag. 124.

*Icon.* Curt. [Curtis 1787]. Bot. Mag. tab. 7.

Bull. [Bulliard 1787]. herb. Tab. 309.

Scopol. [Scopoli 1771] l. c. tab. 3.

Growing in shady places along foothills in southern Europe. [symbol:] perennial herb (seen alive).

This plant has long been generally known as an ornament of gardens due to its early, pretty flowers, and has been described several times.

 

4) *Omphalodes amplexicaulis*. (to me)

Omphalodes with cordate, stem-clasping leaves, the lower ones strongly obtuse, the upper ones slightly acute, glabrous, smooth at the margins, racemes erect with several flowers.

*Cynoglossum lusitanicum*. [Vahl 1791] Symb. bot. 2. p. 34. (excluding synonyms).

*Descript*[*ion*]. Vahl [Vahl 1791] l. c.

**Figure 3.**
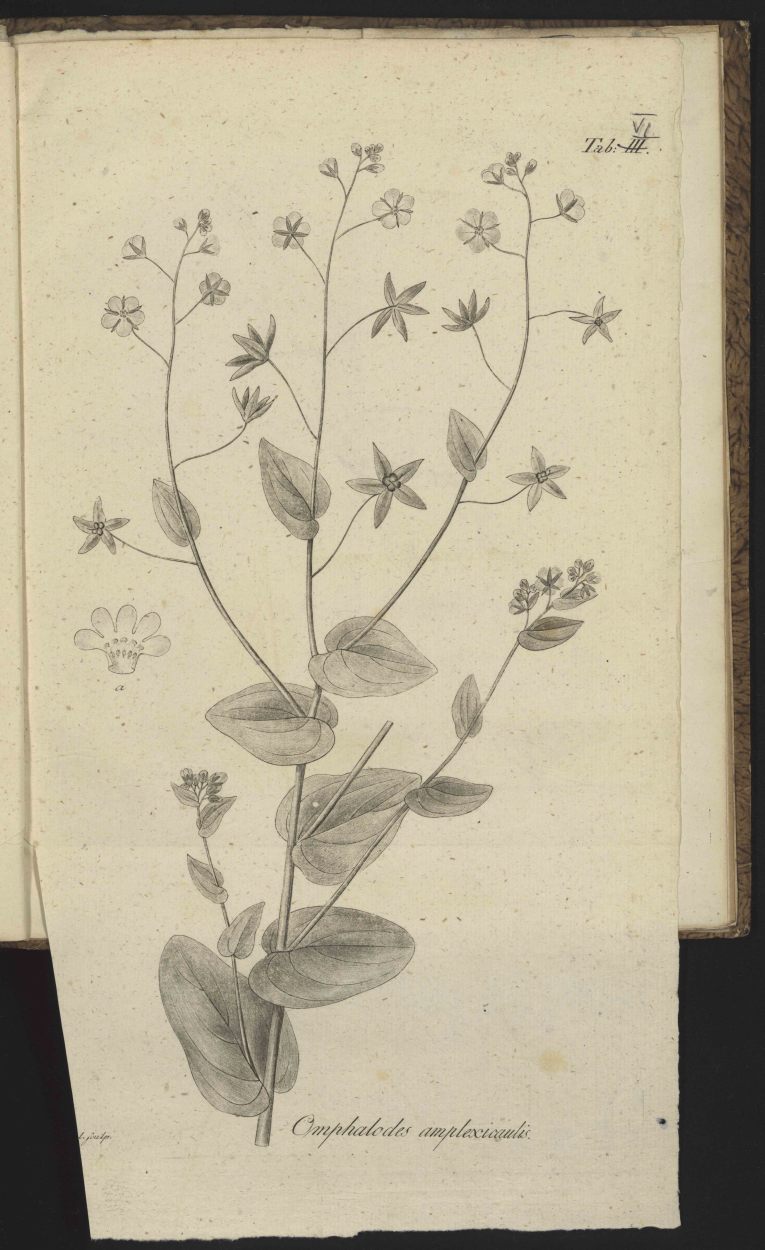
Facsimile of Lehmann 1818 pl. 6.

*Icon.* Plate VI. [Fig. 3].

Habitat [unknown] [symbol:] biennial herb? (dry material seen).

Fig. 3

 

5) *Omphalodes littoralis*. (to me)

Omphalodes with basal leaves spathulate, the cauline ones sessile, oblong, upper ones ovate, widened at base, papillose, strigose at margin, racemes with bracts.

*Descript*[*ion*].

Root perpendicular, absolutely simple. Stem erect, one finger long, glabrous. Leaves papillose, green-glaucous, under the lens [when magnified] with strigose margin; the basal ones spathulate; the cauline ones sessile, oblong, widened at base, one half inch [1.25 cm] long. Racemes axillary and terminal, bracteate. Bracts of the same shape as stem-borne leaves but smaller and more ovate. Pedicels well-spaced, filiform, almost one inch [2.5 cm] long, erecto-patent, finally patent, slightly strigose. Calyces five-parted, with strigose base, and ovate-lanceolate lobes. Corolla as in Omphalodes linifolia. Nutlets four, urceolate, smooth, subglabrous under the lens [when magnified] with hairy base, membrane inflexed, ciliate at margin: cilia white hyaline.

Habitat: occurring on the maritime coasts of western France. [symbol:] biennial herb (dry material seen).

Obs. I. Differs from Omphalodes linifolia: in its lower stature, the shape and width of the leaves; the presence and shape of bracts; and the border of the nutlet membrane that is ciliate but not dentate.

I have seen this plant in several herbaria of French botanists under the name *Cynoglossum linifolium* and have also received it under that name.

 

6) *Omphalodes linifolia*. *Moench*.

Omphalodes with cuneiform basal leaves, stem-borne ones linear-lanceolate, glabrous, with scabrid-denticulate margins, racemes without bracts.

Omphalodes linifolia. Hoffm. et Link [Hoffmannsegg and Link 1811]. Flore portugaise I. p. 193.

Omphalodes linifolia. Moench. [Moench 1794] Methodus p. 419.

*Cynoglossum linifolium* Linn. [Linnaeus 1762] Sp. Plant. I. p. 193.

*Omphalodes lusitanica folio lini*. Tournef. [Tournefort 1700a] Inst. P. 140.

*Icon.* Barrel. [Barrelier 1714] Icon. [. 309] 1234.

Moris. [Morison 1699] Hist. 3. Sect. 11. t. 3. [in fact: 30] f. 11.

Growing in France and Portugal [symbol:] biennial herb (seen alive).

This plant is also generally known, and is grown as an ornamental in most gardens. By cultivation the leaves become two to three times longer and much broader.

 

7) *Omphalodes myosotoides*. (to me)

Omphalodes with basal leaves spathulate-lanceolate, the cauline ones sessile, tuberculate-pilose, rough, flowers loosely racemose.

*Cynoglossum myosotoides*. La Billard. [Labillardière 1791] Plant. Rar. Syriae decas. 2. p. 6.

*Cynoglossum lithospermifolium*. Lamarck [Lamarck 1786] Enc. Bot. vol. 2. p. 240.

*Descript*[*ion*]. La Billard. [Labillardière 1791] l. c.

Lamarck [Lamarck 1786] l. c.

*Icon*. La Billard. [Labillardière 1791] c. Tab. 2.

Growing on the summits of Mount Lebanon [symbol:] perennial herb (dry material seen).

 

8) *Omphalodes scorpioides*. (to me)

Omphalodes with prostrate stem, dichotomous, leaves rough, the basal ones spathulate, the cauline ones lanceolate, sessile, the lower ones opposite, the others alternate, pedicels axillary.

*Cynoglossum scorpioides*. Haenke [Haenke 1789] in Jacq. Collect. Vol. II. Pag. 3.

*Descript*[*ion*] Haenke l. c.

Schmidt. [Schmidt 1794] Fl. Bohem. Cent. III. No. 220^[31]^.

**Figure 4.**
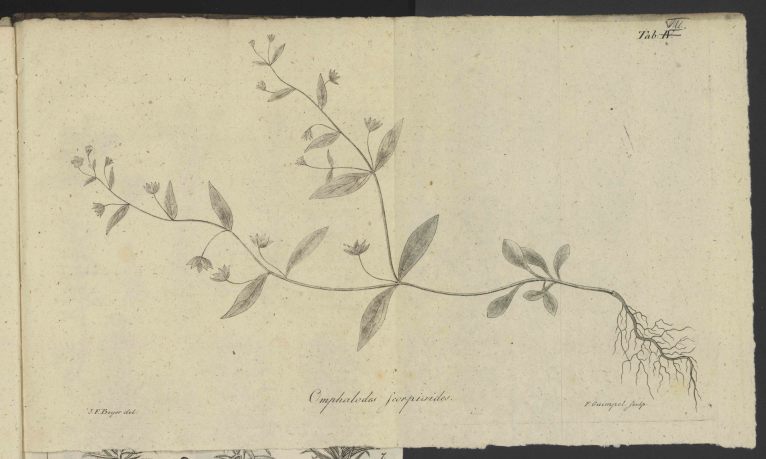
Facsimile of Lehmann 1818 pl. 7.

*Icon*. Plate VII. [Fig. 4]

Habitats are shady wooded areas of Bohemia and apparently also the region of Würzburg, Bavaria. ♂ (seen alive).

Fig. 4

Also most likely belonging to this genus is *Cynoglossum lusitanicum* Linn. [Linnaeus 1762], which I personally am unfamiliar with, and furthermore perhaps *Cynoglossum lusitanicum* Fortis [Fortis 1771].

 

Berlin, in the spring of 1816

 

 

[**11**] ≡ *Pectocarya lateriflora* (Lam.) DC. – Prodr. 10: 120. 1846.

[**12**] = *Pectocarya gracilis* (Ruiz & Pav.) I.M.Johnst. – in Contr. Gray Herb. 70: 36. 1924.

[**13**] *Cynoglossum angustifolium* Willd. – Sp. Pl. 1: 763. 1798.

[**14**] *Cynoglossum emarginatum* Lam. – Tabl. Encycl. 1. 400. 1792.

[**15**] *Cynoglossum racemosum* Schreb. – in Nova Acta Phys.-Med. Acad. Caes. Leop.-Carol. Nat. Cur. 3: 475. 1767 – no online source found.

[**16**] *Cynoglossum cristatum* Schreb. – in Nova Acta Phys.-Med. Acad. Caes. Leop.-Carol. Nat. Cur. 3: 476. 1767 – no online source found.

[**17**] *Cynoglossum echinatum* Thunb. – Prodr. Pl. Cap. 34. 1794.

[**18**] ≡ *Lappula cynoglossoides* (Lam.) Gürke – in Engler & Prantl, Nat. Pflanzenfam. 4(3a): 106. 1894.

[**19**] *Cynoglossum lanceolatum* Forssk. – Fl. Aegypt.-Arab. 41. 1775.

[**20**] *Cynoglossum cheirifolium* L. – Sp. Pl. 1: 134. 1753.

[**21**] *Anchusa lanata*. L. – Syst. Nat., ed. 10: 914. 1759].

[**22**] *Cynoglossum fulvum* Rudolphi – in J. Bot. (Schrader) 1799(2): 279. 1800.

[**23**] *Buglossum* Adans. - Fam. Pl. 2: 178. 1763; not *Buglossoides* Moench - Methodus 418. 1794.

[**24**] Desfontaines refers to *Anchusa officinalis* L. – Sp. Pl. 1: 133. 1753.

[**25**] Either *Cynoglossum montanum* L. – Demonstr. Pl. 5. 1753; **or** *Cynoglossum montanum* Lam. – Fl. Franç. 2: 277. 1779, nom. illeg.

[**26**] [Syst. Veg., ed. 13. 157. 1774].

[**27**] *Cynoglossum rindera* L. f. – Suppl. Pl. 130. 1782.

[**28**] *Mattia* Schult. – Observ. Bot.: 30, 32. 1809 – no online source found.

[**29**] p. 139 “Cynoglossum lusitanicum ist nicht das Linnéische; dieses letztere ist, wie ich von Smith weiß, eine sibirische Pflanze” [*Cynoglossum lusitanicum* is not that of Linnaeus; the latter, as I learn from Smith, is a Siberian plant].

[**30**] According to Dandy and Stearn 1961 Linné’s description of *Cynoglossum lusitanicum* refers to *Omphalodes linifolia*.

[**31**] No online source found.

## Supplementary Material

Supplementary material 1 J.G.C. Lehmann's 'Botanical Observations' of 1818 on Coldenia, Colsmannia, Cynoglossum, and Omphalodes (commented translation) Data type: pdf of original publication
File: oo_5436.pdf Hartmut H. Hilger, Theodor C. H. Cole, Federico Selvi

## Figures and Tables

**Figure 1.**
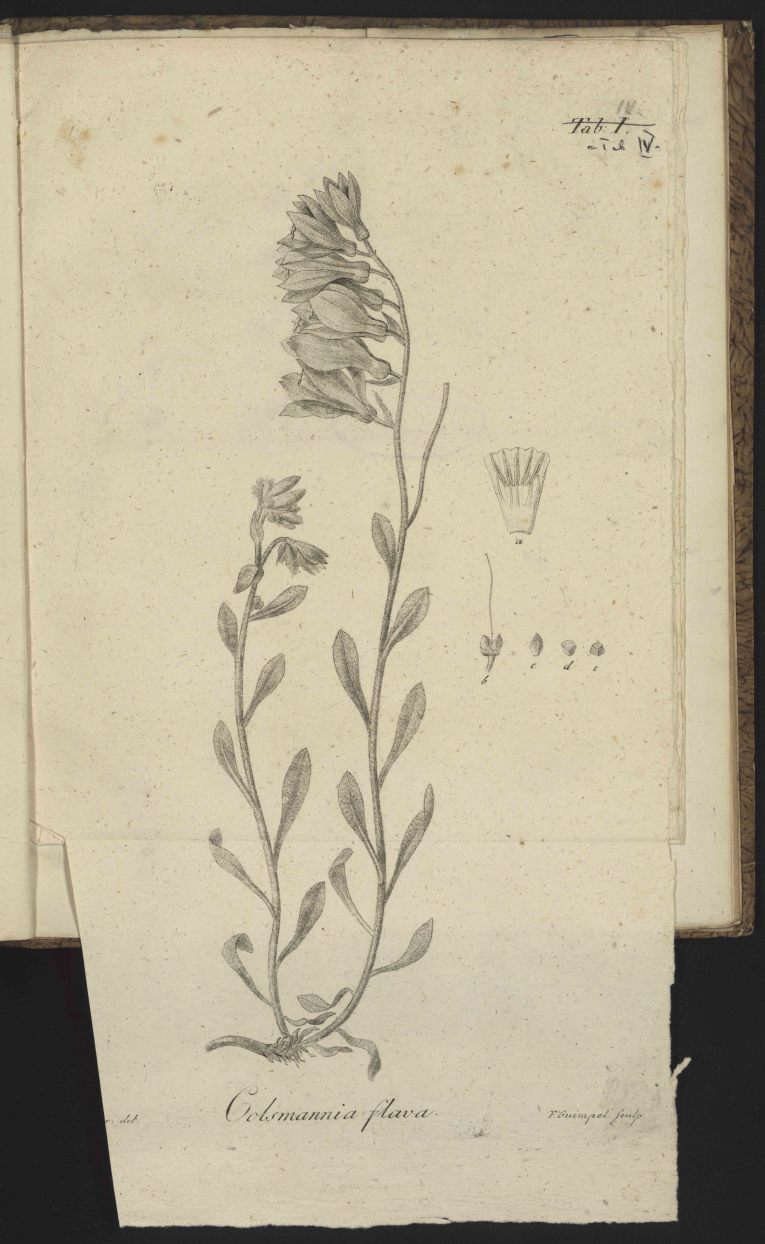
Facsimile of Lehmann 1818 pl. 4.
