# Supplementary material for: J.G.C. Lehmann's 'Botanical Observations' of 1818 on Coldenia, Colsmannia, Cynoglossum, and Omphalodes (commented translation)
Source: Biodivers Data J. 2014 Apr 22;(2):e1064. doi: 10.3897/BDJ.2.e1064 (PMC4031435; doi:10.3897/BDJ.2.e1064)
Supplement: Supplementary material 1 — J.G.C. Lehmann's 'Botanical Observations' of 1818 on Coldenia, Colsmannia, Cynoglossum, and Omphalodes (commented translation) [file biodiversity_data_journal-2-e1064-s001.pdf]

DER

GESELLSCHAFT NATURFORSCHENDER FREUNDE

ZU BERLIN

MAGAZIN

FÜR

DIE NEUESTEN ENTDECKUNGEN

IN DER GESAMMTEN NATURKUNDE.

ACHTER JAHRGANG.

BERLIN 1818,

IN DER REALSCHULBUCHHANDLUNG.

Zweites Quartal 1814

April, May, Juny.

Director:

Fischer, Professor.

IX.

Botanische Beobachtungen.

Von

Dr. *J. G. C. Lehmann.*

Taf. IV. — VII.

COLDENIA.

Diese Gattung gehört zur Pentandria Monogynia unter die nüssetragenden Asperifolien, und steht dem Heliotropium zunächst. Linné rechnete sie zur Tetrandria Tetragynia seines Systems, und an dem Platz, welchen er ihr zuerst angewiesen hatte, ist sie seitdem bei den meisten — selbst bey Willdenow und Persoon — stehen geblieben. Geht man aber bis auf die ersten Ausgaben seiner Species plantarum zurück; so findet man, daſs er selbst diese Pflanze nicht gehörig hatte untersuchen können; denn ausdrücklich setzt er hinzu „Fructificationem ulterius examinent alii in vivente planta, ego perfectam non vidi."

Gärtner schreibt dieser Gattung in seinem Werke: de fructibus et seminibus plantarum Vol. 1. p. 329 vier Staubfäden und einen Staubweg mit zwey Narben zu. Jussieu giebt (Genera plant. pag. 130.) den Gattungscharacter nach Linné an, setzt doch aber schon fragend hinzu: „an Coldenia potius pentandra?" um so mehr, da er eine zweyte Art erhalten hatte, welche nach Dombay's Versicherung einen Kelch und eine Blume mit 5 Einschnitten habe, und dabey zur Pentandria Monogynia gehöre. Die Pflanze deren er erwähnt ist dieselbe, welche von Dr. Persoon. (Synopsis plant. 1. pag. 157.) unter dem Namen Tiquilia dichotoma ist beschrieben worden — das Lithospermum dichotomum der Flora Peruviana Vol. 2. pag. 5. tab. 111.

Daſs diese Gattung Tiquilia zur Coldenia gehöre, zeigt schon der Habitus; daſs Coldenia zu den nüssetragenden Asperifolien gestellt werden müsse, ergiebt sich leicht aus der Vergleichung dieser Pflanze mit der ihr zunächst verwandten Gattung Heliotropium, und ich würde daher Coldenia procumbens nur für eine abnorme Art halten, wenn ich nicht an mehreren in Guinea gesammelten Exemplaren fünftheilige und fünfmännige Blumen selbst gesehen hätte. Nur an den meisten unteren Blumen fehlte der fünfte Theil an Kelch und Corolle, so wie der fünfte Staubfaden, alle aber waren monogyn. — Nuces biloculares, die man der Coldenia procumbens gleichfalls zugeschrieben hat, habe ich auch nie sehen können, sondern allemal vier distincte unten etwas zusammengewachsene Nüsse, wovon zuweilen zwey nicht zur Vollkommenheit gelangen.

COLSMANNIA (Novum genus.)

Character naturalis.

CAL. Perianthium monophyllum, quinquepartitum, campanulatum, petaloideum, maximum, persistens, basi pentagonum: laciniis ovali-lanceolatis patentibus, apice acutis, corolla longioribus.

COR. monopetala, tubulato - campanulata calyce brevior. Tubus cylindricus. Limbus tubuloso — ventricosus, quinquedentatus. Faux nuda pervia.

STAM. Filamenta quinque subcarnosa in tubo corollae. Antherae subulatae sagittatae, in fasciculum pyramidalem collectae, basi liberae, erectae, longitudine filamentorum.

PIST. Germina quatuor. Stylus filiformis corolla longior. Stigma obtusum.

PER. nullum. Calyx inflatus in fundo semina fovens.

SEM. Nuces quatuor, duae oppositae ovatae, triangulares, osseae, nitidae, laeves, receptaculo affixae, basi perforatae, duae saepius abortientes.

Character essentialis.

Calyx quinquepartitus petaloideus maximus basi quinquegonus. Corolla cylindrico — campanulata calyce brevior. Faux nuda. Antherae hastatae liberae. Nuces quatuor triangulares ovatae nitidae osseae perforatae.

Obs. 1) Affinis Onosmati, inprimis quod ad corollam, differt vero: 1) Calyce basi quinquegono, petaloideo, corolla longiori, cujus laciniae ovali — lanceolatae patentes.

2) Antheris basi liberis neutiquam cennexis.
3) Nucibus basi perforatis.

Obs. 2) Species quae characteres hosce genericos exhibuit lecta fuit in Oriente, a Tournefortio ni fallor.

Colsmannia flava. Tab. IV.

Radix brunea, intus alba, superne lignosa ut videtur perennis. Caules plures erecti, simplices, semipedales et ultra, uti tota planta pilis flavis, adpressis, mollibus, densissime obtecti, unde speciei nomen flavae. Folia integerrima, sericea, obovato — lanceolata, obtusiuscula, basi attenuata; inferiora petiolata superiora sessilia, alterna. Inflorescentia: racemus terminalis. Flores pedicellati, ante anthesin nutantes, demum erecti secundi; inferiores bracteis lanceolatis pedicellis longioribus suffulti. Calyx v. s. coloratus pallide flavus, sericeus, interdum quadripartitus; lacinia quarta reliquis duplo major apice bifida. Corolla flava, exteriori facie subpubescens. Stylus purpurascens. Semina nuces v. s.

Fig. *a.* zeigt eine aufgeschnittene Blume von der inwendigen Seite.
Fig. *b.* zwey reife Nüsse in ihrer natürlichen Gröſse und Stellung.
Fig. *c.* eine einzelne Nuſs.
Fig. *d.* den Durchnitt einer Nuſs.
Fig. *e.* die untere Hälfte derselben von unten gesehen.

Durch ihren blumenblattartigen Kelch zeichnet sich diese Gattung von allen andern der ganzen Familie der Boragineen oder Asperifolien besonders aus. Am nächsten kömmt sie, wie ich auch schon angeführt habe der Gattung Onosma, und in dieser dem Onosma sericeum. Bey der Gattung Triplaris, ein Baum aus der Dioecia Decandria, finden wir eine ziemlich ähnliche Bildung des Kelches, nur daſs er so wie die Blume bey jener Pflanze aus 3 Blättern besteht.

Ich habe diese Gattung nach meinem verehrten Freunde dem Herrn Professor Colsmann zu Kopenhagen benannt; der schon durch seine musterhaften Beschreibungen der von Dr. König gesammelten Arten der Gattung Gratiola allen Botanikern bekannt ist, und mehr noch durch seine ausgebreiteten Kenntnisse in allen Fächern der Naturkunde, und durch die seltene Liberalität, womit er die Benutzung seiner vorzüglichen botanischen, entomologischen und mineralogischen Sammlungen, jedem gern gestattet der sich zu unterrichten wünscht, sich die allgemeine Verehrung und Liebe erworben hat.

CYNOGLOSSUM — OMPHALODES.

Diese beyden Gattungen sind sehr wesentlich von einander verschie-

den, sowohl durch das Habituelle, als besonders durch die auffallend verschiedene Bildung der Saamen, welche man bey Omphalodes kaum Nüsse nennen dürfte, wäre es nicht um sie zu der Gruppe der nüssetragenden Asperifolien rechnen zu können. Früher schon getrennt, sind sie später von den meisten Botanikern wieder vereint worden. Es sey mir erlaubt erst einige Bemerkungen über verschiedene Arten der Gattung Cynoglossum hier anzuführen, und dann eine monographische Uebersicht der Omphalodes Arten zu geben.

Cynoglossum lateriflorum. Lamarck Dict. enc. Vol. 2 pag. 239. no. 10, und das *Cynoglossum lineare* Ruiz. et Pavon. Fl. Per. Vol. 2. pag. 6. sind eine und dieselbe Pflanze. Der spätere Name muſs also im System ausgestrichen werden.

Cynoglossum angustifolium. Willd. Sp. plant. T. 1. P. 2. p. 763. Cynoglossum emarginatum. Lamarck Illust. Vol. 1. p. 400. no. 1799. und *Cynogloss. racemosum.* Schreb. in Nov. Act. Nat. Curios. T. II. pag. 475 sind auch eine und dieselbe Pflanze. Schrebers (frühere) Beschreibung weicht nur bey den Saamen etwas von der Willdenowischen ab. Daſs die Lamarckische Pflanze mit hieher gehöre, weiſs ich mit Gewiſsheit, da ich bey meinem Aufenthalte in Paris Originalexemplare gesehen habe; auch führen alle drey dasselbe Synonym aus Tournefort an. Der Schrebersche Name scheint von den neuern Botanikern ganz übersehen worden zu seyn.

Cynoglossum cristatum. Schreber in Nov. Act. Nat. Curios. Vol. III. pag. 476 ist dieselbe Pflanze, welche (später) unter demselben Namen von Lamarck und anderen ist beschrieben worden. Auch hier scheint es hat man die Schrebersche Beschreibung übersehen.

Cynoglossum echinatum. Thunberg. Prodr. Fl. Capens. pag. 34. und Myosotis cynoglossoides. Lamarck Illust. Vol. 1. pag. 396. no. 1778 sind wiederum eine und dieselbe Pflanze. Thunberg hat diese Art späterhin im 3ten Stück, pag. 48. des ersten Bandes von Schraders neuem Journal für die Botanik 1806 ausführlicher beschrieben. Was Lamarck von den Saamen sagt, ist sehr treffend; auch mir scheint diese Art zur Gattung Myosotis zu gehören.

Cynoglossum hirsutum. Thunberg l. c. und *Cynoglossum lanceolatum.* Forsk. Descript. p. 41. sind gleichfalls nicht von einander verschieden.

Cynoglossum cheirifolium. Linn. und *Anchusa lanata.* Linn. sind auch eine und dieselbe Pflanze. Vahl hatte diese Bemerkung bey der Ansicht des Linnéischen Herbariums gemacht, und Hornemann hat sie in einer Enumerat. plant. hort. bot. Hafn. Vol. 1. pag. 177. aufgenommen. Die Pflanze welche Willdenow in seiner Enumeratio pl. hort. bot. Berol.

Vol. 1. pag. 180 unter dem Namen *Cynoglossum cheirifolium* beschrieb, hält Hornemann a. a. O. für eine verschiedene Art, und der gegebenen Characteristik nach scheint es auch so. Durch Fxemplare aus dem hiesigen botanischen Garten, weiſs ich jetzt mit Gewiſsheit, daſs dies nicht der Fall sey, und vermuthe, daſs nur durch einen Schreibfehler von Willdenow der Kelch gröſser (oder wie es doch wol heiſsen soll — länger) als die Blume bezeichnet ward.

Cynoglossum fulvum. Rudolphi in Schraders Journal für die Botanik 2ter Band 1799 4tes Stück pag. 279. *Cynoglossum clandestinum.* Desfont. Fl. Atlant. T. I. pag. 159. tab. 42, und *Cynoglossum officinale.* Brot. Fl. Lusitan. I. pag. 295, sind eine nnd dieselbe Pflanze. (Vergleiche Hoffm. et Link. Flore portugaise l. pag. 190.) Dr. Persoon glaubt (Synops. plant. l. 159.) fälschlich, die Rudolphische Pflanze gehöre zu den Buglossis, und sey der *Anchusa italica* zunächst verwandt. Beyläufig will ich hier bemerken, daſs *Anchusa italica.* Retz. Observ. bot. Fasc. l. pag. 12 von *Anchusa paniculata.* Ait. Hort. Kew. ed. l. pag. 177. nicht verschieden ist und daſs dazu auch noch die *Anchusa officinalis* der Fl. Atlant. l. pag. 157 gehöre.

Cynoglossum Dioscorides. Villars. Fl. Delph. Vol. 2 pag. 457. ist weder eine Abart von *Cynoglossum officinale*, wie Willdenow sp. plant. T. 1. P. 2. pag. 760 meint, noch einerley mit *Cynoglossum montanum* oder *sylvaticum*, wie es allgemein angenommen wird. Ich besitze ein Exemplar, welches mir Villars selbst mitgetheilt hat, und was von *Cynoglossum officinale* sowohl als von *Cynoglossum sylvaticum* ganz nnd gar verschieden ist.

Cynoglossum laevigatum. Linné.

Unter dem Namen *Rindera tetraspis* beschrieb der berühmte Pallas diese Pflanze im ersten Bande seiner Reise, (append. no. 100 tab. 100.) gab ihr aber später in seiner Flora Rossica den Namen *Cynoglossum Rindera*, welchen ihr der jüngere Linné in seinem Supplement pag. 130. beygelegt hatte. Dieser muſs sie also von dem *Cynoglossum laevigatum* seines Vaters für verschieden gehalten haben, denn er beschreibt sie nicht unter demselben Namen, wie Willdenow (Spec. plant. T. I. P. 2. p. 763.) fälschlich anführt. Dieses *Cynoglossum laevigatum* nun ist vom Herrn Schultes (Observ. bot. p. 31.) mit noch einigen andern Arten dieses Geschlechts, abermals zu einer eigenen Gattung vereint worden, der er zugleich den neuen Nameu — Mattia — giebt. Auf den ersten Blick scheint zwar das *Cynoglossum laevigatum* mit *lanatum* und *umbellatum* eine eigne Gattung auszumachen, da sowohl die Blumen als auch die Saamen eine verschiedene Bildung haben. Vergleicht man aber die Blumen und Saamen von Cynoglossum *glastifolium*, *an-*

gustifolium, stamineum, cristatum, laterifforum und noch einigen andern wenig bekannten Arten, welche Herr Schultes vielleicht zu vergleichen nicht Gelegenheit hatte, so wird der Uebergang zu den übrigen Cynoglossis sehr auffallend, und ich glaube daher, daſs jene Arten nicht als eine eigene Gattung angesehen werden dürfen. Will man sie aber durchaus trennen, so lasse man ihnen wenigstens den früheren schon allgemein bekannten Gattungsnamen.

Cynoglossum lusitanicum. Linn. Sp. plant. ed. I. p. 293.
Unter diesem Namen sind nicht weniger als fünf und vielleicht noch mehr ganz verschiedene Pflanzen beschrieben. Linnée's *Cynoglossum lusitanicum* ist, wie wir durch Link, von Smith dem Besitzer des Linnäischen Herbariums wissen, eine sibirische Pflanze; (Vergleiche Schraders neues Journal für die Botanik 1. Band 1806. 3tes Stück pag. 183.) welche, wie wir gleichfalls durch Link erfahren haben, gar nicht in Portugall gefunden wird.

Cynoglossum lusitanicum. Vahl Symb. bot. 2. pag. 34.
Diese Pflanze welche ich unter dem Namen Omphalodes amplexicaulis unten characterisiren werde, wächst gleichfalls in Portugall nicht wild, (Vergleiche Brot. Fl. lusit. Vol. 1. pag. 296) und scheint auch in den Gärten ausgestorben zu seyn. Das Exemplar, welches in der Vahlischen Sammlung aufbewahrt wird, ist aus dem botanischen Garten zu Madrid; vom Dr. Bernades im July 1760 aufgelegt, uud von ihm mit dem Tournefortschen Namen Omphalodes lusitanica elatior cynoglossi folio bezeichnet. Wie man die Characteristik dieser Pflanze:

C. foliis cordatis amplexicaulibus glabris margine laevibus Vahl. l. c.

mit der Linnäischen

C. foliis lineari-lanceolatis scabris.

hat vereinen können, ist mir in der That ganz unbegreiflich.

Cynoglossum lusitanicum. Brot. Fl. lusitan. 1. pag. 296. ist eine von den oben genannten Pflanzen ganz verschiedene Art, welche vom Professor Link unter dem Namen *Omphalodes nitida,* in der Flore portugaise 1. pag. 192 — 94. beschrieben, und tab. 25 prachtvoll abgebildet ist. Willdenow hat diese Art in seiner Enumeratio plant. hort. Berol. Vol. 1. pag. 181. unter den Namen *Cynoglossum nitidum* angeführt.

Cynoglossum lusitanicum. Lamarck Dict. enc. Vol. 2. pag. 239. halte ich nicht für verschieden von *Cynoglossum lusitanicum.* Brot., oder *Omphalodes nitida* Link, und die Lamarckische Beschreibuug, — mehr als seine Diagnose, — im einzelnen passend, wenn auch nicht allemal richtig. Was ich in den Pariser Sammlungen als *Cynoglossum lusitanicum* gesehen habe, war auch mit *Omphalodes nitida* einerley. Herr Professor

Link hält die Lamarckische Pflanze für eine verschiedene Art. (Vergleiche Flore gortugaise 1. pag. 195.)

Cynoglossum lusitanicum. Miller. Dict. no. 6. ist eine bloſse Varietät von Omphalodes linifolia.

Endlich beschreibt der Abbée Fortis in seinen Osservazioni sopra Cherso ed Osero pag. 68. ein *Cynoglossum lusitanicum*, was gewiſs auch noch von allen andern Pflanzen dieses Namens ganz verschieden ist. Da seine Beschreibung kurz und wenig bekannt ist, so halte ich es nicht für überflüſsig sie hier zu wiederholen. „Planta pedalis. Radix lignosa biennis. Folia ovato-lanceolata, integerrima, villosa, ad basin ciliata. Caules laeviter striati, scabri. Flores parvi foliis oppositi, caereleoalbicantes. Semina parva muricata. Fortis l. c.

OMPHALODES. Tournefort. tab. 58. Gärtn. tab. 67. f. 3. C.

Character essentiales.

Calyx profunda 5—fidus. Corolla rotata fauce squamis fornicatis clausa. Nuces 4 depressae stylo oblique incumbentes, margine membranaceae, calathiformes.

1) *Omphalodes nitida. Hoffm. et Link.*

O. foliis oblongo-lanceolatis nervosis supra glabris nitidis subtus pubescentibus, inferioribus longe-petiolatis, superioribus sessilibus.

O. nitida Hoffm. et Link. Fl. portugaise 1. p. 194.

Cynoglossum nitidum. Willd. Enumerat. 1. p. 181.

Cynoglossum lusitanicum. Broter. Fl. Lusit. 1. p. 296.

Cynoglossum lusitanicum. Lamarck Enc. bot. Vol. 2. p. 239.

Omphalodes lusitanica cynoglossi folio. Tournef. Inst. rei herb. p. 140.

Descript. Hoffm. et Link l. c. p. 192 — 95.

Lamarck l. c.

Jcon. Hoffm. et Link. l. c. tab. 25.

Habitat in Lusitaniae locis umbrosis Humidis sylvaticis. ♃. (v. v.)

In unsern Glashäusern blühet diese schöne Pflanze im Anfang des Mai's.

2) *Omphalodes cornifolia.* (mihi)

O. foliis radicalibus longe-petiolatis ovato-cordatis acuminatis nervosis, caulinis subsessilibus infimis lanceolatis summis ovatis, racemis solitariis mnltifloris.

Cynoglossum cappadocicum Willd. Sp. plant. T. 1. P. 2. pag. 767.

Cynoglossum omphalodes. β Lamarck Enc. bot. V. 2. p. 239.

Omphalodes orientalis *corni folio.* Tournefort Cor. p. 7.

Descript.

Planta spithamaea. Caules erecti filiformes, pubescenti-pilosi. Folia integerrima, *supra* subglabra ope lentis hispidula, *subtus* glabra nervis prominentibus alternis pilosiusculis, avenia; *radicalia* longissime-petiolata, ovato — cordata, acuminata, 2 — 4 uncias longa, 1 — 2 uncias lata; *caulina inferiosa* sessilia, oblongo-lanceolata utrinque acuta, breviter-petiolata, semiuncialia; *superiora* vel floralia uncialia, sessilia, ovata, acuta. Flores distantes alterni, ante anthesin nutantes post florescentiam erecti, in racemis terminalibus, solitariis, elongatis, laxis. Pedicelli capillares, unciales et ultra, pilosiusculi. Calyces praesertim versus basin pilosi, quinquepartiti: laciniis ovatis, acuminatis.

Icon. Tab. V.

Habitat in Cappadocia. ♃ (v. s.)

3) *Omphalodes verna. Moench.*

O. foliis radicalibus ovato-cordatis, caulinis ovato-lanceolatis petiolatis subglabris, racemis conjugatis paucifloris.

O. verna Moench. Methodus. pag. 420.

Cynoglossum Omphalodes. Linn. Sp. plant. 1. p. 193.

Descript. Scopol. Fl. Carn ed. 2. 1. pag. 124.

Icon. Curt. bot. mag. tab. 7.
Bull. herb. tab. 309.
Scopol. l. c. tab. 3.

Habitat in locis umbrosis Europae australis ad radices montium. ♃ (v. v.)

Diese Pflanze ist wegen ihrer frühen hübschen Blumen als eine Zierde der Gärten schon lange allgemein bekannt, und auch an mehreren Orten beschrieben.

4) *Omphalodes amplexicaulis.* (mihi)

O. foliis cordatis amplexicaulibus inferioribus obtusissimis superioribus acutiusculis glabris margine laevibus, racemis erectis multi floris.

Cynoglossum lusitanicum. Vahl. Symb. bot. 2. p. 34. (exclusis synonymis.)

Descript. Vahl l. c.

Icon. Tab. VI.

Habitat . . . . ⊙? (v. s.)

5) *Omphalodes littoralis.* (mihi)

O. foliis radicalibus spathulatis caulinis sessilibus oblongis superioribus ovatis basi dilatatis papillosis margine strigosis, racemis bracteatis.

Descript.
Radix perpendicularis, simplicissima. Caulis erectus digitalis, glaber Folia papillosa, glauco-viridia, ad lentem margine strigosa; *radicalia* spathulata; caulina sessilia, oblonga, basi dilatata, semipollicaria. Racemi axillares et terminalis, bracteati. Bracteae forma foliorum caulinorum sed minores et magis ovatae. Pedicelli distantes, filiformes, fere pollicares, erecto-patentes, demum patentissimi substrigosi. Calyces quinque partiti basi strigosi: laciniis ovato-lanceolatis. Corolla ut in O. linifolia. Nuces quatuor urceolatae, laeves, subglabrae ope lentis basi pilosae, membrana inflexa margine ciliata: ciliis albis hyalinis.

Habitat in littoribus Galliae occidentalis. ⊙. (v. s.)

Obs. 1. Differt. a. O. linifolia: statura humiliore; forma et latitudine foliorum; praesentia et forma bractearum; membrana nucularum margine ciliata non vero dentata.

Diese Pflanze habe ich in einigen Herbarien französischer Botaniker unter dem Namen Cynoglossum linifolium gesehen, und auch unter diesem Namen erhalten.

6) *Omphalodes linifolia. Moench.*

O. foliis radicalibus cuneiformibus caulinis lineari-lanceolatis glabris margine denticulato-scabris, racemis ebracteatis.

O. linifolia. Hoffm. et Link. Flore portugaise 1. p. 193.
O. linifolia. Moench, Methodus p. 419.
Cynoglossum linifolium Linn Sp. plant. 1. p. 193.
Omphalodes lusitanica folio lini. Tournef. Inst. p. 140.
Icon. Barrel. Icon. 1234.
Moris. Hist. 3. Sect. 11. t. 3. f. 11.

Habitat in Gallia et Lusitania. ⊙. (v. v.)

Auch diese Pflanze ist allgemein bekannt, und wird gleichfalls zur Zierde in den meisten Gärten gezogen. Durch Cultur erhält sie zwey bis dreymal längere und viel breitere Blätter

7) *Omphalodes myosotoides.* (mihi).

O. foliis radicalibus spathulato - lanceolatis caulinis sessilibus lineari-lanceolatis tuberculoso-pilosis asperis, floribus laxe-racemosis.

Cynoglossum myosotoides. La Billard. Plant. rar. Syriae decas. 2. p. 6.
Cynoglossum lithospermifolium. Lamarck Enc. bot. Vol. 2. p. 240.
Descript. La Billard. l. c.
Lamarck l. c.
Icon. La Billard l. c. Tab. 2.

Habitat in summo cacumine montis Libani. ♃ (v. s.)

8) *Omphalodes scorpioides.* (mihi)

O. caule prostrato dichotomo, foliis scabris radicalibus spathulatis caulinis lanceolatis sessilibus inferioribus oppositis reliquis alternis, pedicellis axillaribus.

Cynoglossum scorpioides. Haenke in Jacq. Collect. Vol. II. pag. 3.
Descript. Haenke l. c.
Schmidt. Fl. Bohem. Cent. III. no. 220.
Icon. Tab. VII.

Habitat in locis nemorosis umbrosis udis Bohemiae, inque Episcopatu Herbipolitano. ♂ (v. v.)

Zu dieser Gattung gehört denn wahrscheinlich noch das mir unbekannte Cynoglossum lusitanicum Linn. und vielleicht auch Cynoglossum lusitanicum Fortis.

Berlin im Frühling 1816.

X.

Botanische Beobachtungen

von

Curt Sprengel *).

Tab. VIII. IX.

SCABIOSA ELEGANS. Spreng. pug. 2. n. 48.

Sc. corollulis quinquefidis radiantibus, anthodio hirto flores aequante, foliis oblongis amplexihaulibus inciso-serratis nitidis utrinque villosiusculis, caule ramoso.

Das Vaterland dieser schönen Art ist mir unbekannt. Ich erzog sie 1809

*) Wir bemerken, daſs ausser den dieser Abhandlung wirklich beigegebenen Tafeln VIII und IX. auch Scabiosa elegans und caucasica, Sonchus caucasicus und Santolina crithmifolia auf besondern Tafeln abgebildet von dem würdigen Verf. eingeschickt worden sind. Ob wir nun zwar gern einräumen, daſs diese Abbildungen wesentlich zu den Beschreibungen, und an diese gewissermassen gebunden sind, so konnten wir doch wegen der dem Herrn Verleger schuldigen Verbindlichkeit die Tafeln (besonders die illuminirten) nicht noch mehr als wirklich geschehen ist, häufen. Ja, wir konnten nicht anders als unter den zur gegenwärtigen Abhandlung gehörenden Abbildungen eine Auswahl in denjenigen Tafeln treffen, welche zu Phylica und Salvinia gehören, weil uns diese von verhältniſsmäſsig ganz vorzüglicher Wichtigkeit und Nothwendigkeit zu seyn schienen, wogegen wir die übrigen Abbildungen nachliefern werden, sobald es künftig der Raum gestattet, und nachdem die zur Abhandlung über die Blattwespen (Gattungen Pterygophorus und Hylotoma) im 6ten Jahrgange des Magazins p. 276. u. f. gehörenden zwey Tafeln, welches nächstens geschehen wird, nachgeliefert worden sind.
Die Gesellsch. naturf. Fr.

~~Tab. I.~~ Tab. IV.

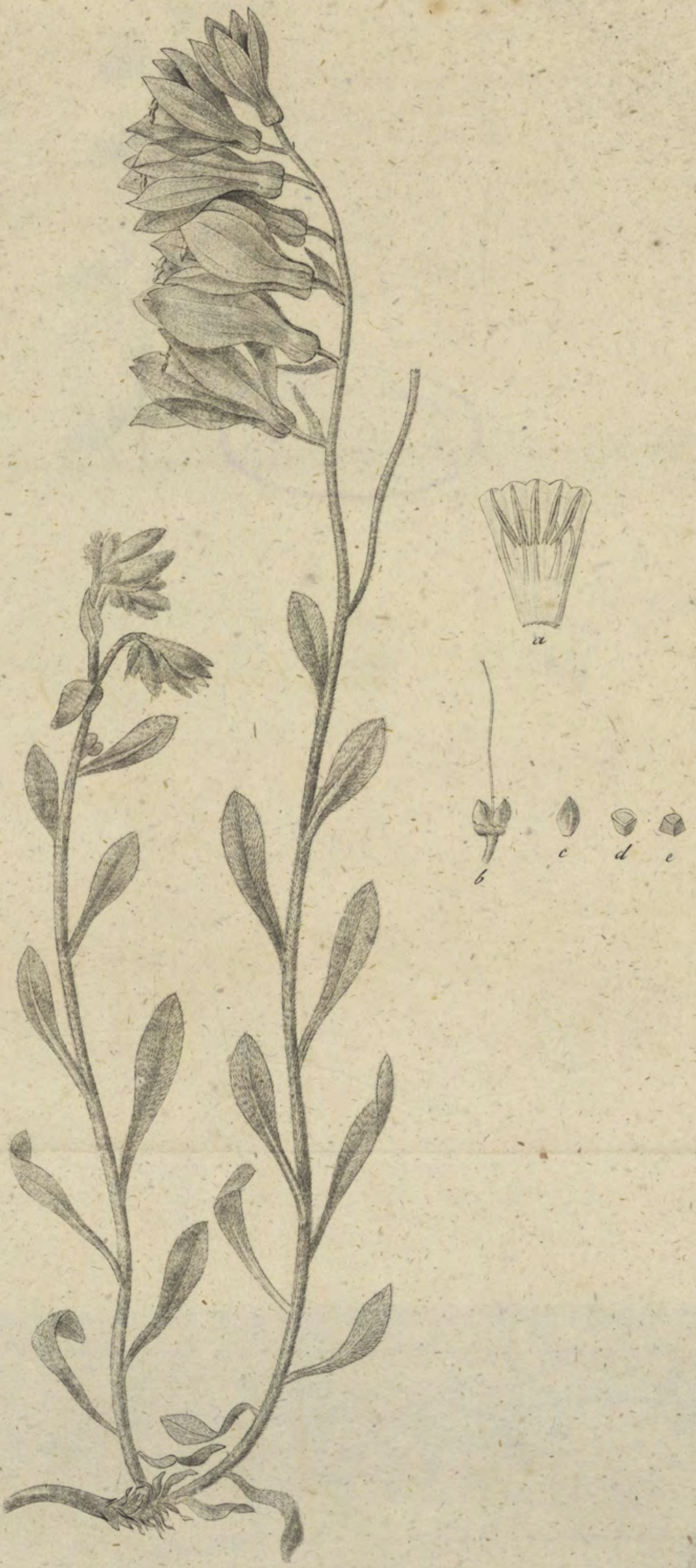

Colsmannia flava.

[illegible]r. del. F. Guimpel sculp.

Tab: ~~III~~. VI.

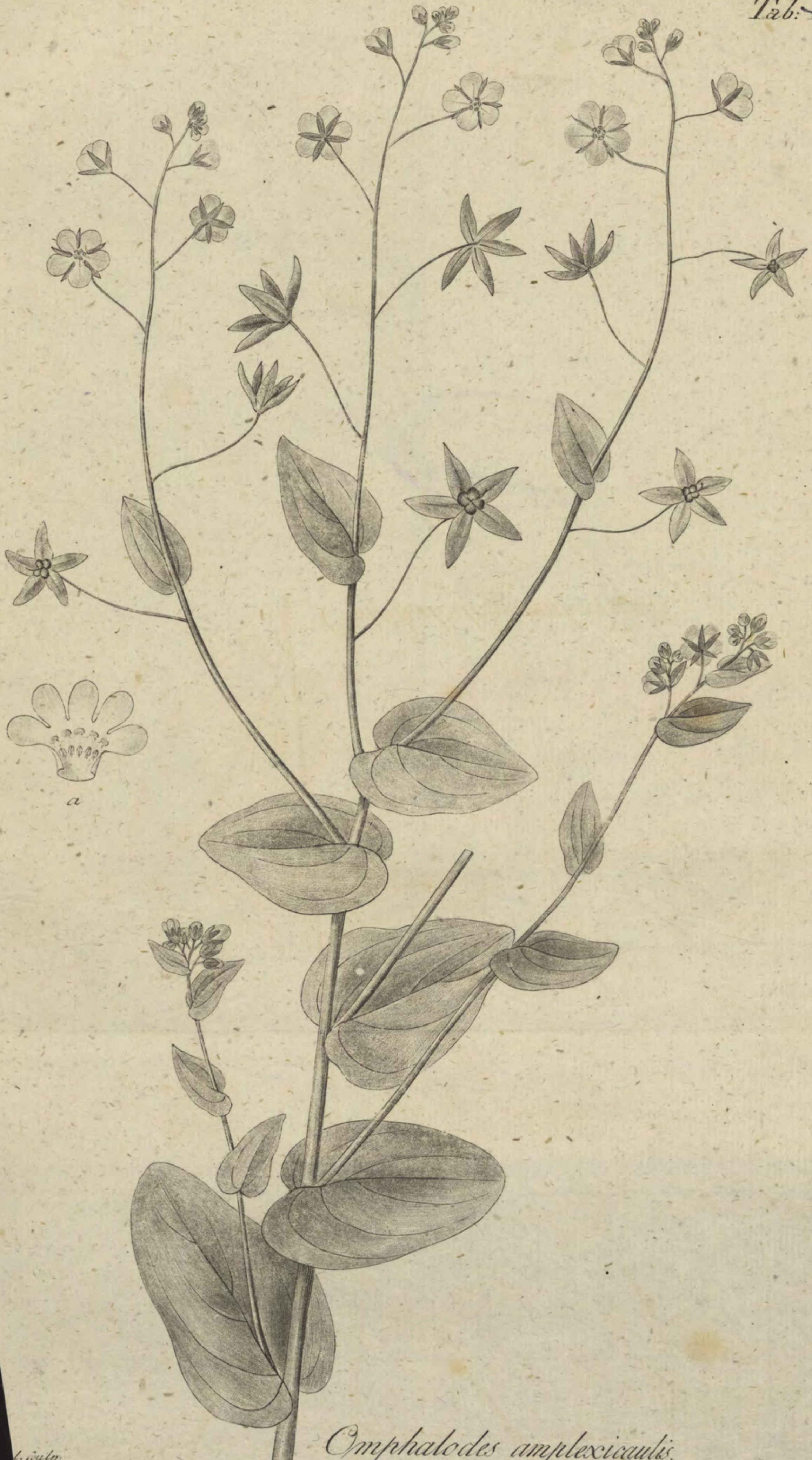

[illegible]l. sculp.

Omphalodes amplexicaulis.

Tab. ~~II~~ =V

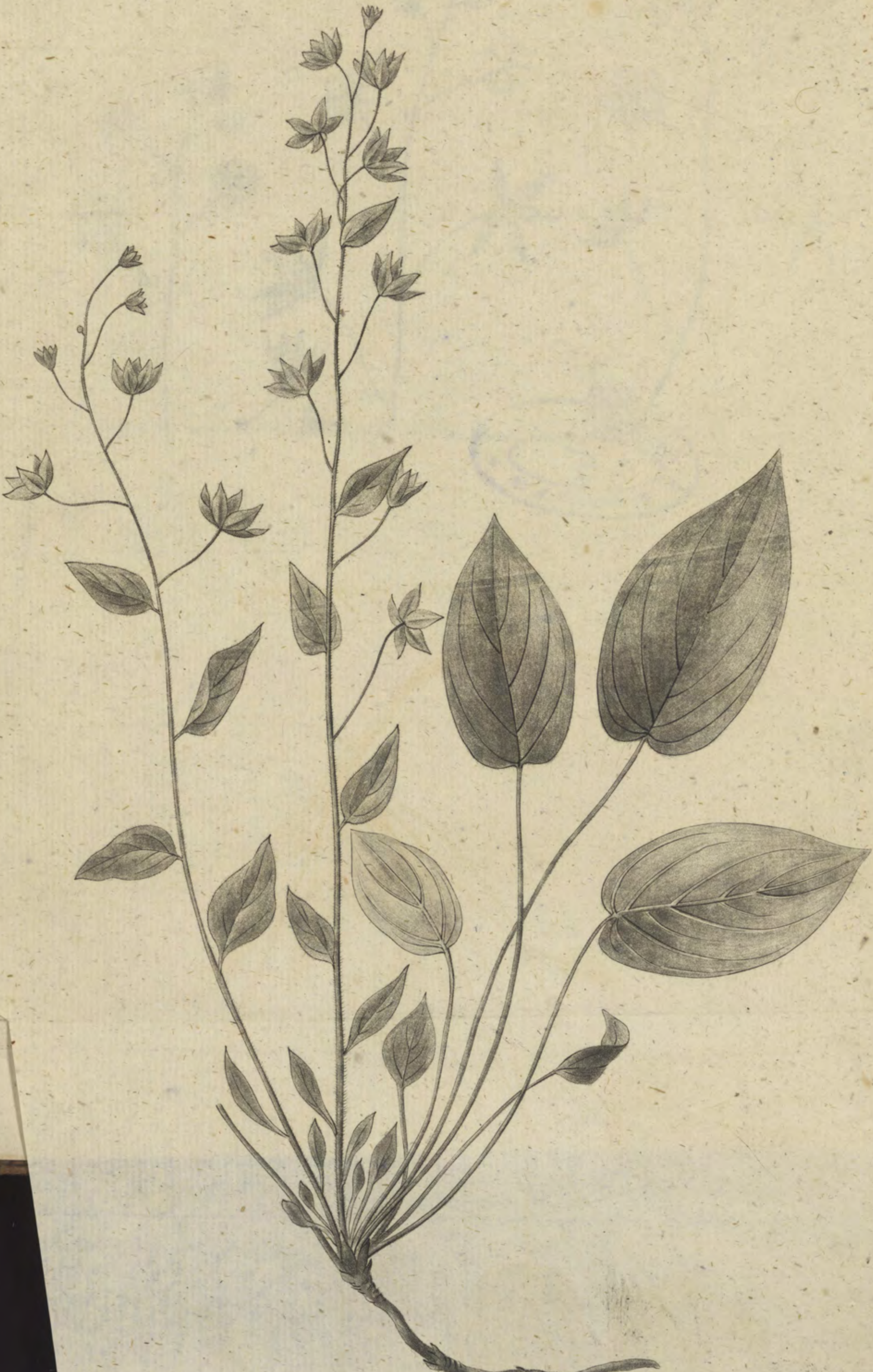

del: *Omphalodes cornifolia* F. Guimpel. sculp.

Tab: ~~IV~~ VIII.

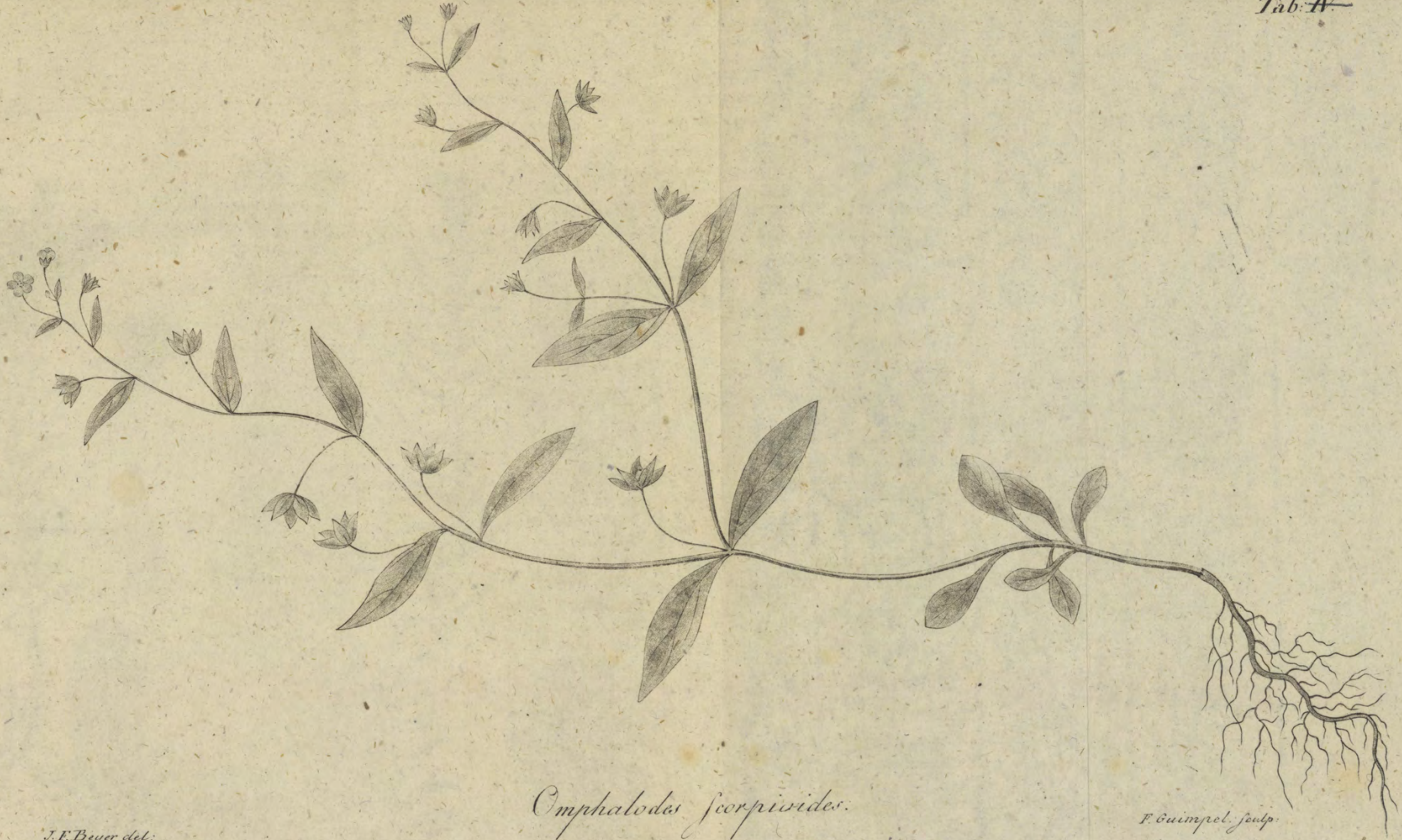

Omphalodes scorpioides.

J. F. Beyer del:

F. Guimpel. sculp:
